# Healthcare Utilization with Drug Acquisition and Expenses at the National Health Insurance Fund in Sudan

**DOI:** 10.3390/healthcare10040630

**Published:** 2022-03-27

**Authors:** Yasir Ahmed Mohammed Elhadi, Abdelmuniem Ahmed, Ramy Mohamed Ghazy, Elhadi B. Salih, Osman S. Abdelhamed, Ramy Shaaban, Hammad Mohamed Hammad Mohamed, Alanood Elnaeem Mohamed, Noha Ahmed El Dabbah, Ashraf Ahmed Zaher Zaghloul

**Affiliations:** 1 Department of Health Administration and Behavioral Sciences, High Institute of Public Health, Alexandria University, Alexandria 21561, Egypt; nohaeldabbah@alexu.edu.eg (N.A.E.D.); hiph.azaghloul@alexu.edu.eg (A.A.Z.Z.); 2 Department of Public Health, Medical Research Office, Sudanese Medical Research Association, P.O. Box 382, Khartoum 11111, Sudan; 3 Physiology Department, Faculty of Medicine, University of Gezira, Wad Madani 2667, Sudan; monem33246@hotmail.com; 4 Tropical Health Department, High Institute of Public Health, Alexandria University, Alexandria 21561, Egypt; ramy_ghazy@alexu.edu.eg; 5 Federal Ministry of Health, Khartoum 11111, Sudan; elhadi2811@hotmail.com; 6 Teaching Assistant, Faculty of Medicine, Ahfad University for Women, P.O. Box 167, Omdurman 14415, Sudan; osmansalahe@hotmail.com; 7 Department of Instructional Technology and Learning Sciences, Utah State University, Logan, UT 84321, USA; ramy.shaaban@usu.edu; 8 Al-Mana General Hospital, Al-Jubail 35718, Saudi Arabia; hammad.adam4@yahoo.com; 9 Faculty of Pharmacy, University of Science and Technology, Khartoum 11111, Sudan; alanood.alnaeem@gmail.com

**Keywords:** out-of-network utilization, health insurance, drug acquisition, medication costs, National Health Insurance Fund, Sudan, catastrophic health expenditure

## Abstract

Background: Understanding the pattern of care use can provide valuable information for reform interventions. This study investigates the pattern of healthcare utilization and its association with drug acquisition patterns and expenses in the National Health Insurance Fund (NHIF) of Al Jazira State in Sudan. Methods: A cross-sectional survey was conducted at NHIF primary healthcare centers of Al Jazirah state in Sudan. Results: A total of 768 beneficiaries were interviewed, of which 63.2% reported using out-of-network physician care, while 36.8% receive care from the NHIF physician network only. More than half (60.8%) of NHIF-interviewed clients reported a heavy burden of medication costs. The pattern of physician utilization was significantly associated with the number and source of regular drugs, the burden of out-of-pocket payment, and monthly out-of-pocket expenditures on medications, (*p* < 0.001). The regression analysis revealed that gender, marital status, number of chronic diseases, and number of regular drugs were the significant predictors of the pattern of physician care utilization; these factors explained nearly 36% of the variance in respondents’ pattern of physician care utilization. Conclusions: An impressive proportion of out-of-network care was found in Al Jazirah State in Sudan. The NHIF stakeholders should consider medication subsidy as a potential strategy for decreasing patient leakage to out-of-network services.

## 1. Introduction

Many developing countries are debating whether to implement Compulsory Health Insurance Schemes (CHIS) [1]. Health insurance schemes are increasingly being recognized as a tool for financing health care provision in developing countries, with the potential to increase utilization, better protect people from catastrophic health expenses, and address equity issues [2]. The satisfaction of clients with services provided is one of the primary difficulties facing social health insurance in developing countries [3]. The National Health Insurance Fund (NHIF) is a governmental organization that was established in Sudan in 1995 [4]. According to the NHIF statistical report of 2021, the coverage of NHIF reached 81.7% of the Sudanese population. However, several studies have found that NHIF members receive poor quality care in Sudan and other Sub-Saharan African countries [5,6,7,8,9]. Patients’ views of service quality, in particular, influence their confidence and subsequent behavior in terms of selecting and using accessible healthcare services [8].

The conceptualization and measurement of using out-of-network care differ among studies. Generally, out-of-network use of health services results when patients within a health system’s designated population receive care from providers outside of that system. Guirguis et al. (2003) addressed different categories of out-of-network utilization described in the literature; these included (1) utilization of healthcare services that are neither covered by the plans nor paid for by them, (2) utilization of healthcare services covered by the plans that insurance plan members obtained from outside-plan providers and the plan paid, e.g., emergency services and referrals to outside plan providers, and (3) utilization of healthcare services covered by health insurance plans but the beneficiaries chose to obtain from outside-plan providers and the plan did not pay [10]. 

According to Kyanko et al. [11], there are two types of out-of-network care: voluntary choice and involuntary use of an out-of-network physician, and both are influenced by three domains: patient, encounter, and system-related factors. When patients are unaware of their provider’s network status at the time of service, this is considered involuntary out-of-network care. On the other hand, when individual preferences are considered in selecting a healthcare provider outside of the health insurance coverage network, it is referred to as voluntary out-of-network care. The latter differs in that the beneficiary decides to get health services from outside-plan providers although the service is accessible in-network and would have been covered at a lower or no cost. Seeking voluntary out-of-network care includes a dimension of client satisfaction with their health plans. 

The detrimental influence of out-of-network utilization of care upon both the health insurance plans and beneficiaries has been a major issue for policymakers because it has been studied since the past century [12,13,14]. It affects health insurance plans in terms of reduced technical efficiency, in addition to catastrophic costs incurred by the beneficiaries of health plans. Catastrophic health expenditure (CHE) means “that medical spending of a household exceeds a certain level of capacity to pay” [15]. There is no agreement on what level of healthcare spending is considered catastrophic [16]. However, the WHO defines it as out-of-pocket expenditure that surpasses 40 percent of total household income minus basic expenses [17].

To date, researchers continue to describe different patterns, categories, and determinants of seeking out-of-plan care in many countries around the globe [18,19,20,21]. Guirguis et al. [10] investigated the extent of out-of-plan services use among clients attending Health Insurance Organization (HIO) clinics in Egypt. The prevalence estimate for out-of-plan health services was 70.7%, with beneficiaries using at least one out-of-plan health service every year, according to their findings. Another study in the United States (US) reported a 69% incidence of out-of-network care among adolescent Health Maintenance Organization enrollees [22]. A recent study among patients with rheumatoid arthritis (RA) enrolled in the US Veterans Affairs (VA) healthcare system indicated that 6% of respondents reported having a non-VA rheumatologist [18].

Understanding the pattern of care use can provide valuable information for reform interventions in health insurance schemes. However, unavailable data on the pattern of healthcare utilization or its associated factors among insured clients in Sudan was the trigger to this study. This study aimed to investigate the pattern of physician care utilization and its association with drug acquisition patterns and expenses in the NHIF of Al Jazira State in Sudan. 

## 2. Materials and Methods

This study presented findings from a baseline comprehensive survey that explored the magnitude and determinants of out-of-network primary health care utilization among an insured population under the NHIF in Sudan

### 2.1. Study Design and Setting

A cross-sectional survey was conducted at NHIF primary healthcare centers, Direct Services Provision Centers (DPCs) of Al Jazirah state in Sudan. The NHIF has eight primary DPCs spread across seven districts in Al Jazirah state. Al Jazirah is one of Sudan’s 18 states. The state is located in the country’s east-central portion, between the Blue and White Niles. It has a total land area of 27,549 km^2^. The population count of Al Jazirah state is 5 million, ranked second after Khartoum, with a population density of 179/km^2^. 

### 2.2. Population and Sampling

#### 2.2.1. Population

The target population consisted of insured clients aged 18 and older who attended NHIF primary care clinics in Al Jazirah state. The current study included only patients who visited a general practitioner (GP). Patients complaining of debilitating illnesses visiting specialists or consultants were excluded from this survey. 

#### 2.2.2. Sampling

According to the NHIF data report from 2021, the total estimated number of beneficiaries in the state of Al Jazirah was 3,568,079. The minimum required sample size of 768 clients was calculated using the Epi info 7 software, a statistical software for epidemiology developed by Centers for Disease Control and Prevention in Atlanta, Georgia. The sample size was calculated based on the following assumptions: 50% expected magnitude for out-of-network physician visit use, design effect was equal to 2, alpha error of 0.05, and the margin of error was 5%. The study comprised all the eight DPCs of the NHIF in Al Jazirah state. Based on average monthly visits, the proportional allocation was used to establish the required number of beneficiaries at each of the DPCs. Rooms assigned to GPs were listed to form a sampling frame; each day the investigators selected the rooms randomly using folded papers. Patients examined in the selected rooms were invited to participate in the study. The eight DPCs visited sequentially; when the required number of beneficiaries was reached, the investigators shifted to the next clinic. Interviews were conducted on Saturday, Monday, and Thursday. 

### 2.3. Study Variables and Data Collection

The dependent variable for this study was the pattern of physician visit utilization (in-network vs. out-of-network). Out-of-network physician utilization was defined in the current study as a physician’s visit obtained from outside the NHIF physician network during the previous six months of the interview. In this case, the beneficiary decided not to use the NHIF facilities though the service was accessible and would have been covered. Participants were coded as out-of-network users = 0 and NHIF users only = 1, where out-of-network users represented NHIF clients who reported seeking out-of-network physician care during the six months previous to the interview and NHIF users only represented clients seeking care only within the NHIF physicians’ network during the six months previous to the interview. The independent variables include participants’ sociodemographic characteristics; namely, age, gender, marital status, number of dependents, chronic diseases, and drug acquisition patterns (all from NHIF, part out-of-pocket, or all out-of-pocket), and monthly out-of-pocket expenses on medications. 

Data was collected using a structured interview schedule [10] from SeptemberߝOctober 2021 using KoBo Toolbox, software developed by the Harvard Humanitarian Initiative for mobile data collection in challenging field settings [23].

### 2.4. Data Analysis

Data was fed to the computer and analyzed using IBM SPSS software package version 20.0 (Armonk, NY, USA: IBM Corp) [24]. 

The Kolmogorov–Smirnov test was used to verify the normality of the distribution of variables. Descriptive statistics were given using the N (%) format. We carried out a bivariate analysis to examine variables significantly associated with pattern care utilization. Multivariate logistic regression analysis was carried out to detect the significant predictors of out-of-network users. The significance of the obtained results was judged at the 5% level of alpha error. The R software was used to build a nomogram plot for the significant predictors of the pattern of care utilization. 

## 3. Results

### 3.1. Socio-Demographic Factors Associated with the Pattern of Care Utilization

Table 1 shows the socio-demographic characteristics of respondents based on their pattern of care utilization (in-network or out-of-plan). Of the 768 NHIF clients interviewed (mean age 46.4 ± 13.1 years, 55.1% females), more than three-fifths (63.2%) had used out-of-plan physician visits in the six months before the interview. There was statistically significant difference between out-of-network users and NHIF users only in all the estimated socio-demographic factors (*p* < 0.001).

### 3.2. Association of the Pattern of Physician Care Utilization with Drug Acquisition and Expenses

The pattern of drug acquisition among out-of-network users and NHIF users only is shown in Table 2. More than half of NHIF users only had no regular medications (64.3%), yet the percentage of participants who reported taking four or more combined medications was higher than out-of-network users (5.3% vs. 2.3%). Nearly a third of out-of-network users have been prescribed either one or two regular drugs (31.5% and 32.2% respectively), which is higher than NHIF users only, with only 10.6% and 15.5% reported regularly taking one or two classes of medications, respectively. Concerning the source of regular drugs, roughly 93.1% of out-of-network incurred part out-of-pocket expenses on medications compared to about 75.2% of the NHIF users only. Drug unavailability at NHIF clinics as the main cause for part/all out-of-pocket expenditure on medications was reported equally by out-of-network users and NHIF users only, at 58.2% and 59.5%, respectively. The median out-of-pocket expenses on drugs were higher among NHIF users (4000 vs. 3000). The pattern of physician care utilization was significantly associated with the number of regular drugs (*p* < 0.001), source of regular drugs (*p* < 0.001), the burden of out-of-pocket payment (*p* < 0.007), and monthly out-of-pocket expenditures on medications (*p* < 0.001) (Table 2). 

### 3.3. Logistic Regression Analysis

**Figure 1:**
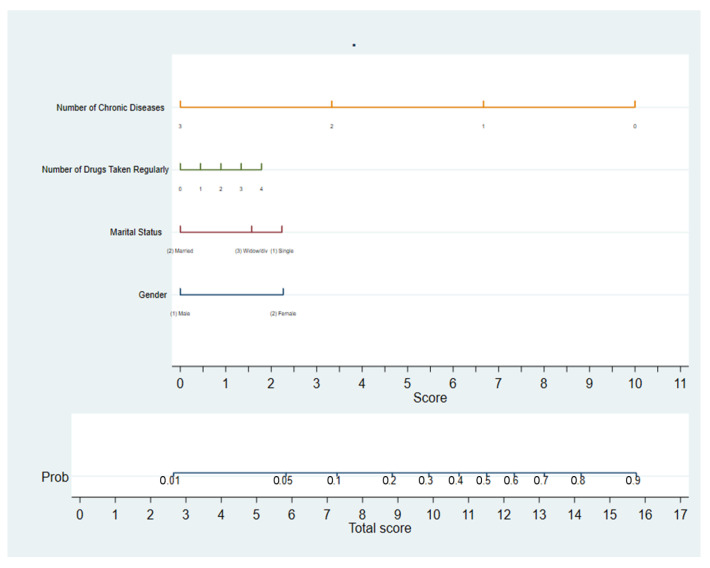
Nomogram plot for the significant predictors of using out-of-network care among NHIF insured clients in Al Jazirah state.

Table 3 shows the results of logistic regression analysis for the predictors of using out-of-network care in the NHIF of Al Jazirah State in Sudan. Females were more than two times more likely to be out-of-network users compared to males (OR = 2.65, 95%C.I (1.76–3.99), *p* < 0.001). Compared to married participants, single clients were more likely by two times to use out-of-network care (OR = 2.3, 95% C.I (1.28–4.12), *p* < 0.05), while divorced/widowed participants were nearly two times more likely to be out-of-network users (OR = 1.91, 95% C.I (1.11–3.30), *p* < 00.19). On the other hand, participants having two or more than three chronic diseases were less likely to be out-of-network users (OR = 0.27, 95% C.I (0.09–0.77), *p* < 0.02 and OR = 0.19, 95% C.I (0.04–0.84), *p* < 0.028 respectively). Adjusting for all possible confounders, participants having 1–3 regular drugs were less likely to be out-of-network users than those with no regular medications prescribed (ORs <0.1, <0.26, <0.13, respectively, *p* < 0.001). These factors explained 36% of the variance in respondents’ patterns of physician care utilization. The probability of using out-of-network care according to the significant predictors in the model is shown in Figure 1. 

## 4. Discussion

Sudan is a low-middle-income country. It spends about 6.5% of its gross domestic product (GDP) and 8.2% of its general government expenditure on health [25]. The main sources of general government health expenditures are federal (5.49%) and state (20.84%), while the total private health expenditure represents 73.14% of the total health expenditures (THE). Of the THE, 70% is out-of-pocket and translates to 84.24 USD per capita, which points to low government expenditure on health [26]. Pharmaceutical expenditure accounts for 2.2% of the GDP and makes up 36% of the total health expenditure [27]. Compared to other countries in the Middle East and North Africa (MENA), pharmaceutical spending ranges between 0.36% and 3.47% of GDP and between 11% and 49.3% of health expenditure in the MENA countries. Pharmaceutical spending as a proportion of health expenditure is highest in Lebanon (49.3%), Jordan (33.8%), and Algeria (31.2%) [28].

The results of the study on the pattern of care utilization, medication acquisition, and expenses among beneficiaries under the National Health Insurance Fund of Sudan indicated that more than half of the insured population of Al Jazirah state have used at least one out-of-network service during the sixth months prior to the interview, while more than a third of them seek physician care only within NHIF. Seeking the available, covered in-network services from outside the network conveys beneficiaries’ dissatisfaction with the quality of health care services. In a recent review of the pros and cons of national health insurance, the authors provided a SWOT analysis indicating that the poor quality of care provided at NHIF in Sudan represents a major threat to NHIF [5]. Stakeholders at NHIF should consider quality improvement projects to provide quality care that meets customer expectations. On the other hand, a study among VA clients in the US that included patients with RA concluded that almost all VA clients rely almost exclusively on VA for RA care and acquisition of both synthetic and biological disease-modifying antirheumatic medicines [18].

This current study highlights the financial burden of the catastrophic medication costs incurred by NHIF beneficiaries in Sudan. More than half (60.8%) of NHIF-beneficient interviewed clients reported a high burden of medication prices, as most people in Sudan (58% of housholds) cannot afford a daily food basket [29]. Rising prescription drug costs pose a significant challenge to many healthcare systems [30]. Previous research has shown that out-of-pocket costs can constitute a financial barrier, reducing the uptake of preventive services and medications [31]. This problem is not exclusive to health insurance systems in developing countries. Sam Caldbeck et al. investigated the financial burden of out-of-pocket prescription drug expenses in Canada and concluded that a vulnerable group of ‘working poor’ are likely to encounter significant financial load since they are not candidates for governmental welfare programs [32]. Moreover, a study in Mexico concluded that self-expenses on medication were the most prevalent component of health expenditures [33]. Patients who cannot afford their prescriptions may reduce their dosage, take them every other day, or stop taking them entirely. Cost-related noncompliance has been linked to an increased risk of financial insecurity, disease progression, hospitalization, and death [34]. Lucero-Prisno et al., (2020) [35] highlight the recent catastrophic medication crisis in Sudan amid COVID-19. They provided an in-depth analysis of the root causes that contributed to medication shortage, unaffordability, and uncontrolled prices. They relate the problems to the economic meltdown and political instability Sudan has been experiencing amid the COVID-19 pandemic. 

Results from the current study revealed that gender is a significant predictor of the pattern of care utilization. Females are two times more likely to seek out network care. According to previous studies, gender differences exist in the pattern of care utilization, with female adults using more healthcare than males, particularly for GP visits [36,37,38]. Women use significantly more services and incur significantly higher out-of-pocket costs than men [39,40]. In support of our findings, some research on the pattern of care utilization has shown that the female gender is a significant predictor for seeking out-of-network physician care [10,22]. 

The marital status and number of chronic diseases were also significant predictors of physician care utilization. Unmarried beneficiaries were more likely to go for out-of-network care. This finding was supported by recent data from Medicare Current Beneficiary Survey, which showed that married beneficiaries use in-network outpatient services at a higher rate than unmarried beneficiaries [41]. The number of chronic diseases was a significant predictor in characterizing the pattern of care utilization. Participants who had more than two chronic diseases were less likely to use out-of-network care. The same results were reported among HIO enrollees in Egypt [10]. However, previous research indicated that multimorbidity is significantly associated with higher out-of-pocket expenditures on medications [42]. 

This study showed that the number of medications used was an important determinant affecting care utilization. In comparison to participants with no regular medications, those with 1–3 regular drugs were less likely to be out-of-network users. This could be explained by the effect of the NHIF medication subsidy for network members in Sudan. The NHIF in Sudan is providing a 25% discount on total medication costs to members. It is likely that NHIF beneficiaries rely on this medication subsidy and attend the NHIF for the sake of more affordable medicine costs and to avoid the high and uncontrolled drug prices in Sudan. It has been documented that medication cost is a major driver of out-of-pocket payment among the insured in developing and developed countries [43,44,45]. On the other hand, research on out-of-network physician care utilization among beneficiaries of HIO in Egypt concluded that there was no significant effect of the number of regular drugs used on the pattern of care utilization (in-network or out-of-network) [10]. 

### 4.1. Implications for Policy and Practice

This report is presented for consideration as a policy document that suggests a need for assessment regarding out-of-network service utilization in the NHIF of Sudan. The study directs the attention of policy leaders toward seeking a means to update or formulate health laws and mandates to subsidy health services and medications, develop different co-payment policies, and expand the availability of medications for the NHIF network members.

### 4.2. Strength and Limitations

This study is the first in Sudan to assess the pattern of care utilization, medication acquisition, and expenses among beneficiaries under the National Health Insurance Fund. The tool used for conducting the survey is a valid reliable tool, and this greatly affects internal consistency. One of the main limitations of this survey was that we excluded patients with chronic diseases visiting health facilities (specialist and consultant clinics) for their higher severity of illness, but this category absolutely cannot be ignored. Second, this study was conducted in Al Jazirah, one of the largest states in Sudan; however, larger studies with different contexts may yield different outcomes. Finally, the nature of the adopted study design (cross-sectional survey) has its own limitations. The fundamental disadvantage of the cross-sectional research design is that, while exposure and result are measured concurrently, there is no indication of a temporal link between exposure and outcome. It is impossible to establish a real cause-and-effect relationship without longitudinal data.

## 5. Conclusions

More than half of insured NHIF clients are out-of-network users. Clients in Al Jazirah state in Sudan incur catastrophic medication costs and suffer a heavy financial burden. The medication subsidy is among the factors that might reduce the clients‘ leakage to out-of-network services. The NHIF stakeholders should consider medication subsidies as a potential strategy for decreasing patient leakage to out-of-network services. Increasing the medication subsidy might have a dramatic effect on reducing out-of-network leakage in the NHIF of Al Jazirah state in Sudan. 

## Figures and Tables

**Table 1:** Socio-demographic factors associated with the pattern of physician care utilization.

Socio-Demographic Variables	The Pattern of Physician Care Utilization				Total (*n* = 768)		*p*-Value
	Out-of-Network Users (*n* = 485)		NHIF Users only (*n* = 283)				
	No.	%	No.	%	No.	%	
**Age**							
18–<40	99	20.4	131	46.3	230	29.9	<0.001 *
40–	126	26.0	69	24.4	195	25.4	
50–	150	30.9	38	13.4	188	24.5	
60+	110	22.7	45	15.9	155	20.2	
Mean ± SD.	49.2 ± 11.9		41.7 ± 13.7		46.4 ± 13.1		
**Gender**							
Male	251	51.8	94	33.2	345	44.9	<0.001 *
Female	234	48.2	189	66.8	423	55.1	
**Marital status**							
Married	319	65.8	132	46.6	451	58.7	<0.001 *
Single	47	9.7	76	26.9	123	16.0	
Divorced/Widowed	119	24.5	75	26.5	194	25.3	
**Number of dependents**							
Less than 3	88	18.1	93	32.9	181	23.6	<0.001 *
Three or more	397	81.9	190	67.1	587	76.4	
**Chronic diseases**							
None	113	23.3	180	63.6	293	38.2	<0.001 *
one	154	31.8	46	16.3	200	26.0	
Two	189	39.0	43	15.2	232	30.2	
Three or more	29	6.0	14	4.9	43	5.6	

* Statistically significant at *p* < 0.05

**Table 2:** Association of the pattern of physician care utilization with drug acquisition and expenses.

Drug Acquisition and Expenses	The Pattern of Physician Care Utilization				Total (*n* = 768)		*p*-Value
	Out-of-Network Users (*n* = 485)		NHIF Users only (*n* = 283)				
	No.	%	No.	%	No.	%	
**Number of drugs taken regularly**							
0	110	22.7	182	64.3	292	38.0	<0.001 *
1	153	31.5	30	10.6	183	23.8	
2	156	32.2	44	15.5	200	26.0	
3	55	11.3	12	4.2	67	8.7	
4+	11	2.3	15	5.3	26	3.4	
**Source of regular drugs**	(*n* = 375)		(*n* = 101)		(*n* = 476)		
All NHIF	7	1.9	17	16.8	24	5.0	<0.001 *
Part out-of-pocket	349	93.1	76	75.2	425	89.3	
All out-of-pocket	19	5.1	8	7.9	27	5.7	
**Reason for out-of-pocket regular use**	(*n* = 368)		(*n* = 84)		(*n* = 452)		
Drug not dispensed by NHIF^®^	214	58.2	50	59.5	264	58.4	0.881
The amount dispensed by NHIF is not enough	97	26.4	20	23.8	117	25.9	
Irregular attendance at NHIF clinics	57	15.5	14	16.7	71	15.7	
**The burden of regular out-of-pocket drugs**	(*n* = 368)		(*n* = 84)		(*n* = 452)		
Heavy	212	57.6	63	75.0	275	60.8	0.007 *
Reasonable	141	38.3	17	20.2	158	35.0	
Not a burden	15	4.1	4	4.8	19	4.2	
**Monthly out-of-pocket expenditure on drugs (SDG)**	(*n* = 368)		(*n* = 84)		(*n* = 452)		
1000–5000	291	79.1	46	54.8	337	74.6	<0.001 *
5000–10,000	67	18.2	25	29.8	92	20.4	
10,000+	10	2.7	13	15.5	23	5.1	
Median (IQR)	3000 (3000–4000)		4000 (3000–6000)		3500 (3000–5000)		

* Statistically significant at *p* < 0.05; SDG: Sudanese Pounds.

**Table 3:** Logistic regression analysis for the predictors of using out-of-network care in the NHIF of Al Jazirah State in Sudan.

Predictors	OR (95% C.I of OR)	*p*-Value	aOR (95% C.I of aOR)	*p*-Value
**Age**				
<40 ^®^	–	–	–	–
40–	0.41 (0.28–0.61)	<0.001 *	0.88 (0.54–1.44)	0.614
50–	0.19 (0.12–0.3)	<0.001 *	0.62 (0.35–1.10)	0.103
60+	0.31 (0.20– 0.46)	<0.001 *	0.83 (0.41–1.66)	0.589
**Gender**				
Male ^®^	–	–	–	–
Female	2.16 (1.590–2.925)	<0.001 *	2.65 (1.76–3.99)	<0.001 *
**Marital status**				
Married ^®^	–	–	–	–
Single	3.90 (2.58–5.93)	<0.001 *	2.30 (1.28–4.12)	0.005 *
Divorced/Widowed	1.52 (1.07–2.17)	0.020 *	1.91 (1.11–3.30)	0.019 *
**Number of dependents**				
Less than 3 ^®^	–	–	–	–
Three or more	0.45 (0.32–0.64)	<0.001 *	0.97 (0.59–1.61)	0.918
**Chronic diseases**				
None ^®^	–	–	–	–
one	0.19 (0.13–0.28)	<0.001 *	0.60 (0.21–1.68)	0.327
Two	0.14 (0.10–0.21)	<0.001 *	0.27 (0.09–0.77)	0.015 *
Three or more	0.30 (0.15–0.60)	0.001 *	0.19 (0.04–0.84)	0.028 *
**Number of drugs taken regularly**				
0 ^®^	–	–	–	–
1	0.12 (0.08–0.19)	<0.001 *	0.10 (0.05–0.19)	<0.001 *
2	0.17 (0.11–0.26)	<0.001 *	0.26 (0.13–0.49)	<0.001 *
3	0.13 (0.07–0.26)	<0.001 *	0.13 (0.05–0.33)	<0.001 *
4+	0.82 (0.37–1.86)	0.641	1.90 (0.58–6.34)	0.284

Model R^2^ = 0.36 OR: Odds ratio. aOR: Adjusted odds ratio C.I: Confidence *: Statistically significant at *p* ≤ 0.05. ^®^: reference value.

## Data Availability

The questionnaire and associated data used in the current study are available from the corresponding author upon reasonable request.
